# A Brief Overview on Antioxidant Activity Determination of Silver Nanoparticles

**DOI:** 10.3390/molecules25143191

**Published:** 2020-07-13

**Authors:** Zdenka Bedlovičová, Imrich Strapáč, Matej Baláž, Aneta Salayová

**Affiliations:** 1 Department of Chemistry, Biochemistry and Biophysics, Institute of Pharmaceutical Chemistry, University of Veterinary Medicine and Pharmacy, Komenského 73, 041 81 Košice, Slovakia; imrich.strapac@uvlf.sk (I.S.); aneta.salayova@uvlf.sk (A.S.); 2 Department of Mechanochemistry, Institute of Geotechnics, Slovak Academy of Sciences, Watsonova 45, 040 01 Košice, Slovakia; balazm@saske.sk

**Keywords:** silver nanoparticles, antioxidant activity, silver nanoparticles synthesis

## Abstract

Our objective in this review article is to find out relevant information about methods of determination of antioxidant activity of silver nanoparticles. There are many studies dealing with mentioned problem and herein we summarize the knowledge about methods evaluating the antioxidant activity of silver nanoparticles reported so far. Many authors declare better antioxidant activity of silver nanoparticles compared to the extract used for synthesis of them. In this review, we focused on methods of antioxidant activity determination in detail to find out novel and perspective techniques to solve the general problems associated with the determination of antioxidant activity of silver nanoparticles.

## 1. Introduction

Nanoscience and nanotechnology have been introduced as interdisciplinary fields in biology, chemistry, physics, and bioengineering. Nanoparticles, in general, are particles of size 1–100 nm and are considered extremely small as the term “nano” means dwarf (from Greek νάννος) in the meaning of extremely small [1,2]. Unique chemical, physical, and biological properties lead to broad range of applications in electronics, sensors, spectral analysis, catalysis, or pharmaceutical industry in finding new ways of drug synthesis [3].

Antimicrobial properties are typical for silver and its compounds including silver nanoparticles (AgNPs) [4]. In view of the mentioned fact, the AgNPs are studied as antibacterial agents to inhibit resistant strains by multiple mechanisms of action, involving induction of oxidative stress, inhibition of DNA replication, or interaction with enzymes and proteins [5,6]. On the other hand, silver nanoparticles have a potential in the treatment of cancer [7,8] or degenerative Alzheimer’s disease because of their antioxidant properties [9]. For example, well characterized PVP-coated AgNPs were used in A549 cells (human lung cancer cells) to study reactive oxygen species production leading to programmed cell death [10]. Another study published by Lee et al. [11] was focused on cytotoxic activity in normal and cancer cell lines. They also showed significant cytotoxicity against A549 cells caused by green synthesized AgNPs from water extract of *Annemarrhena asphodeloides*. These nanoparticles also involved an increase in oxidative stress in A549 cells and inhibition of cell migration, but it is important to say that AgNPs do not exhibit significant toxicity to normal cell lines (3T3-L1 pre adipocyte cell lines) [11].

Antioxidant properties are currently extensively studied for various materials, including the natural ones, in order to identify new compounds from natural sources. The aim of this review is to provide a brief overview on antioxidant activity of silver nanoparticles, its measurement, and future perspectives in relationship between AgNPs and antioxidant activity.

## 2. Antioxidant Activity

### 2.1. Oxidative Stress

**Figure 1:**
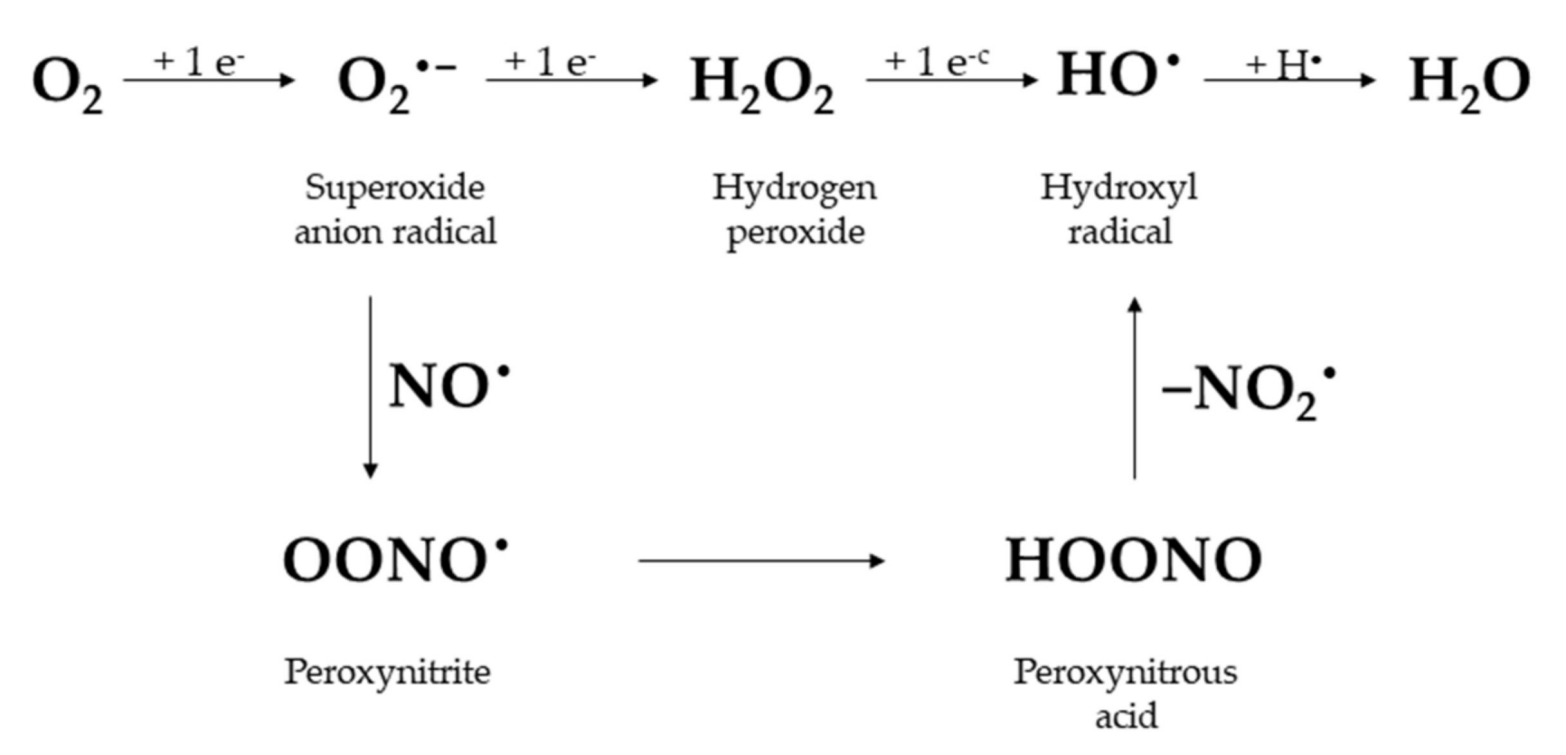
Some reactive oxygen and nitrogen compounds and relationships between them, redrawn from Greguška [15].

Oxidative stress is a phenomenon that can be defined as a state when the equilibrium between the antioxidative defense of cell and oxidants is disrupted by the effect of excess of the oxidants, for example reactive oxygen or nitrogen species (ROS or RNS, respectively) and organic compounds containing sulphur producing alkyl sulfanyl radicals (RS•). For example, transition metal ions at their lower oxidation states are not oxidant species by themselves, but may provoke the formation of ROS or RNS by reacting with hydrogen peroxide or molecular oxygen, thereby serve as prooxidants. Of course, also the presence of the oxidants leads to oxidative modifications of biological system on molecular level (unsaturated bonds of lipids, proteins, DNA, etc.,) causing damage and finally, cellular death is accelerated [12]. This phenomenon occurs when oxidative substances are excessively formed or accumulated and defense mechanisms have failed. Reactive oxygen species represent the most important group of oxidants containing radicals (hydroxyl •OH, superoxide ion $O_{2}^{•-}),$ as well as non-radicals (hydrogen peroxide, organic peroxides) [13]. Superoxide and hydroxyl radical are products of oxygen reduction by electrons (Figure 1) [14,15].

From biological point of view, superoxide anion radical $O_{2}^{•-}$ is generated by the mitochondrial respiratory chain and phagocytic NADPH oxidase (nicotinamide adenine dinucleotide phosphate oxidase; NO_X_), so it is produced by respiration and component part of defense system [16]. It is known that human body produces around 5 g of ROS per day. These are with dynamic balance with the production of any oxygen forms essential for living and protection against the toxic influence of ROS [17]. In addition, an excessive amount of reactive nitrogen species is formed during the oxidative stress. The most common representative of RNS is nitroxide (NO•) produced by nitrogen oxidation catalyzed by NO-synthase [18]. NO• is able to rapidly react with reactive oxygen species (mainly with superoxide anion radical) and produce further reactive nitrogen compounds, for example peroxynitrite or peroxynitrous acid which can be further transformed to NO_2_• or •OH (Figure 1). These products may initiate new radical reactions leading to damage of biomolecules (nitrosylation of DNA or proteins) [19].

The action of ROS and RNS leads to an oxidation of double bond of polyunsaturated fatty acids in lipids (lipoproteins, membrane structures) and formation of aldehydes or peroxides and a result of these processes is changing the membrane permeability [20,21]. The effect of oxidative stress on proteins leads to changes of ion transport (mainly Ca^2+^ ions homeostasis), protein inactivation, and enzymatic activity modification [22]. DNA is damaged by deoxyribose ring cleavage, base modification or chain breaks leading to mutations, translation errors or inhibition of proteosynthesis [23]. According to the mentioned facts, there is a connection between oxidative stress and several diseases caused by oxidative stress [24], e.g., Alzheimer’s disease [25], atherosclerosis [18], cardiovascular diseases [20], cancer [26,27,28], but also psychic impairments, such as ADHD or schizophrenia [29,30].

Oxidative stress is induced by many generally known factors, including chemical, physical, or biological ones, and silver nanoparticles are no exception. Several studies described the cytotoxic effect of AgNPs through ROS generation [31,32,33]. On the other hand, the production of ROS, can be decreased by pre-treatment of cells by NAC (*N*-acetylcysteine) as a systematic antioxidant [33].

It is necessary to note that oxidative stress has not only negative effects on human body, as free radicals have an irreplaceable function in living organisms. Definitely, one of the most important biological mechanism in which free radicals play an important role is the process of phagocytosis including the defense against pathogenic microorganisms [34].

Regarding the cell damage at molecular level caused by oxidative stress, the cell produces some intracellular or extracellular compounds serving as antioxidant defense systems.

### 2.2. Antioxidants

Antioxidants represent a form of opposition to oxidants. Antioxidants are natural or synthetic substances that may prevent or delay damage of cell caused by oxidants (ROS, RNS, free radicals, other unstable molecules) [12]. Halliwell and Gutteridge defined antioxidant as any substance that delays, prevents, or removes oxidative damage to a target molecule [35]. In order for the substance to be considered as an antioxidant, it must be active at low concentration (phenolic antioxidants often loose activity at high concentration and act as prooxidant), its amount needs to be satisfactory high to deactivate the target molecule, it must react with oxygen or nitrogen free radicals, and the final product of the reaction should be less toxic than removed radical. There is no universal antioxidant, as different antioxidants react with different reactive species by various mechanisms, at various locations and protect specific molecular targets [35,36]. Generally, the antioxidant defense can become active either by in vivo processes (synthesis of intracellular enzymes—superoxide dismutases, superoxide reductases, peroxiredoxins, glutathione peroxidases, catalases, peptides—glutathione; or in the form of extracellular antioxidant defenses—synthesis of transferrin, erythrocytes, albumin, urate, glucose; low-molecular mass agents—bilirubin, *α*-keto acids, melatonin, lipoic acid, coenzyme Q, uric acid) or by supplying missing substances in the form of a diet (vitamins—C, E, A, D, riboflavin, thiamine, niacin, pyridoxine, carotenoids, flavonoids, polyphenols, amino acids, folic acid, phytoalexins, elements Se, Fe, Zn, Mg) [35,37,38].

## 3. Antioxidant Activity Determination Methods

Determination of antioxidant activity (or capacity) of samples of various origin is based on different methodologies and assays. The principle of antioxidant capacity lies in chemistry, from which it was adapted to other scientific areas such as biology, medicine, nutrition [39,40]. In simple words, the antioxidant capacity describes the ability of molecules to scavenge free radicals [41]. In Table 1, we provide an overview of methods for antioxidant capacity evaluation and some of them are briefly described [12,42,43,44].

### 3.1. Spectrometric Methods

In recent years, a broad range of spectrophotometric assays has been developed to measure antioxidant capacity. These methods are based on the reaction of colored radical or complex with the antioxidant molecule capable of donating a hydrogen atom. The appropriate standard (Trolox or ascorbic acid) is applied for quantification of antioxidant capacity as trolox equivalent antioxidant capacity (TEAC) or ascorbic acid equivalent antioxidant capacity (AEAC). In this part of our review we would like to pay attention to some methods which apply either AEAC or TEAC.

**Scheme 1:**
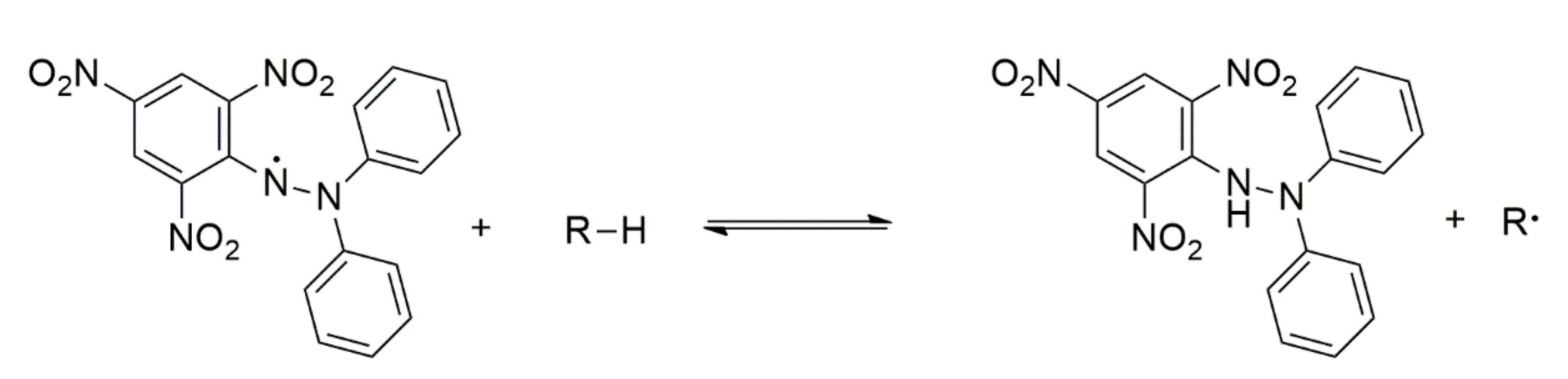
Reaction of DPPH radical with hydrogen donor [46].

Among the most frequently used methods for determining antioxidant capacity are the ABTS and DPPH assays. DPPH• (2,2-diphenyl-1-picrylhydrazyl) is a purple stable free radical reacting with hydrogen donor (Scheme 1). The presence of delocalized spare electrons on the whole molecule prevents dimerization and also gives the color to the molecule of DPPH with absorption maximum at around the value of 520 nm in UV/Vis spectra. The DPPH• radical after reaction gives the reduced form DPPH (hydrazine form), which results in the color change from purple to pale yellow. The level of disappearance of purple color depends on the concentration of the antioxidant. The scavenging capacity is usually determined in organic solvents, not in aqueous media [12,39,41,42,45,46].

It is a rapid, simple, and an inexpensive method for antioxidant capacity determination, but it has some limitations. The first is that DPPH radical can interact with other radicals and the time response curve is not linear with different ratio of antioxidant and DPPH [47]. Some problems may be observed in quantitative analysis because of interference of absorbance with compounds present in sample [48]. Finally, the DPPH method (as all the spectrometric methods) is unsuitable for emulsions since it reflects the partitioning of antioxidants and is also unsuitable for samples containing proteins which precipitate in alcohol [46].

**Scheme 2:**
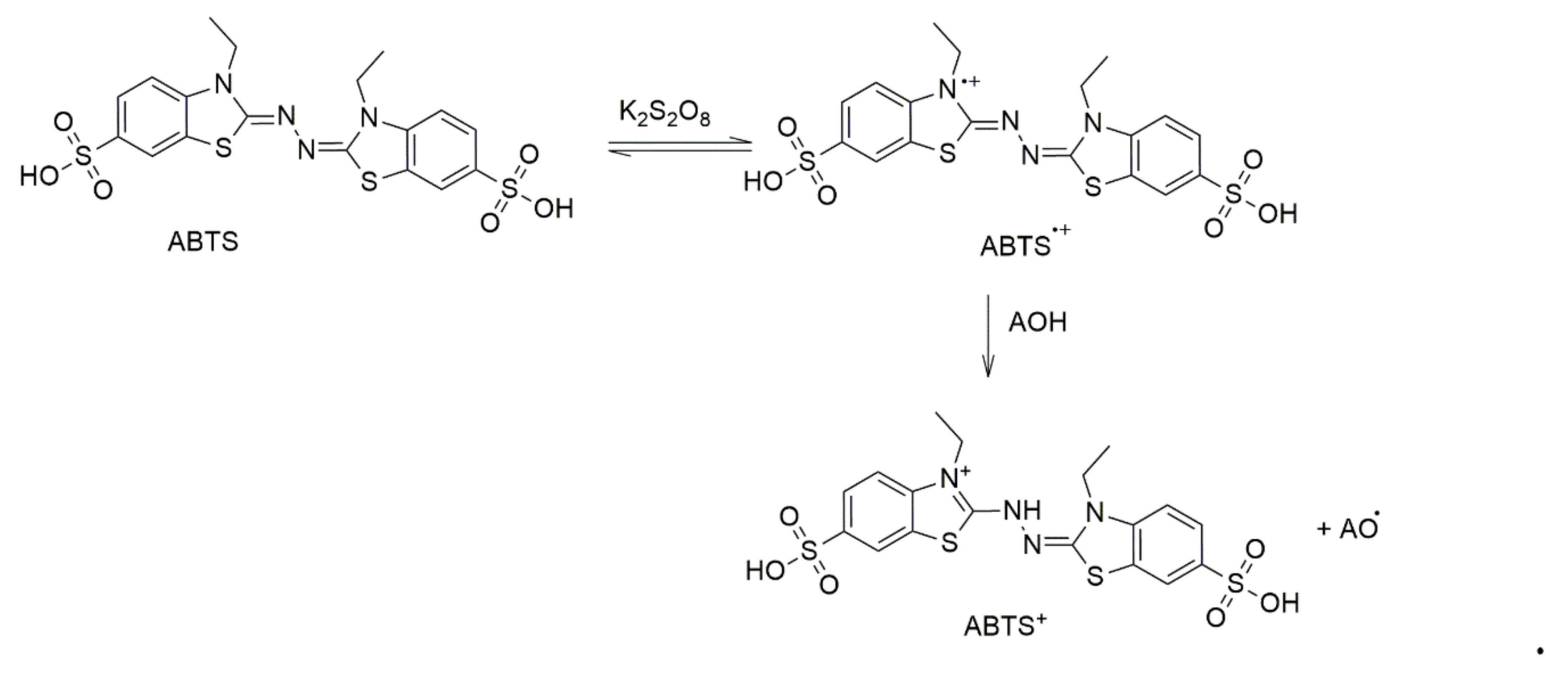
Generating of ABTS^•+^ and its reaction with an antioxidant [52].

The ABTS (2,2′-azino-bis-3-ethylbenzthiazoline-6-sulphonic acid) method or the TEAC (trolox equivalent antioxidant capacity) is based on generating a cation radical ABTS•^+^ formed by emitting one electron from the nitrogen atom (Scheme 2). ABTS is usually first oxidized by potassium persulphate [49], manganese dioxide [50], or AAPH (2,2′-azobis-(2-amidino-propane) dihydrochloride) [51] which gives rise to the cation radical ABTS^•+^. It absorbs at 414, 645, 734, and 815 nm and gives blue-green color. The preferred wavelength is 734 nm because the interference with other absorbing components is minimized. The ABTS^•+^ reacts with antioxidant leading to the decolorization of a solution in the range of 1–30 min [12,39,41,42].

The use of ABTS^•+^ radical has the advantage over the DPPH radical as it can be used in both aqueous and organic media [48]. ABTS method is also useful in studying the effect of pH on the antioxidant activity of various compounds [53]. However, the ABTS assay has some reservations in the overall applications, such as specificity for reaction of different antioxidants, storage of the radical, or processing method conditions [53].

**Scheme 3:**
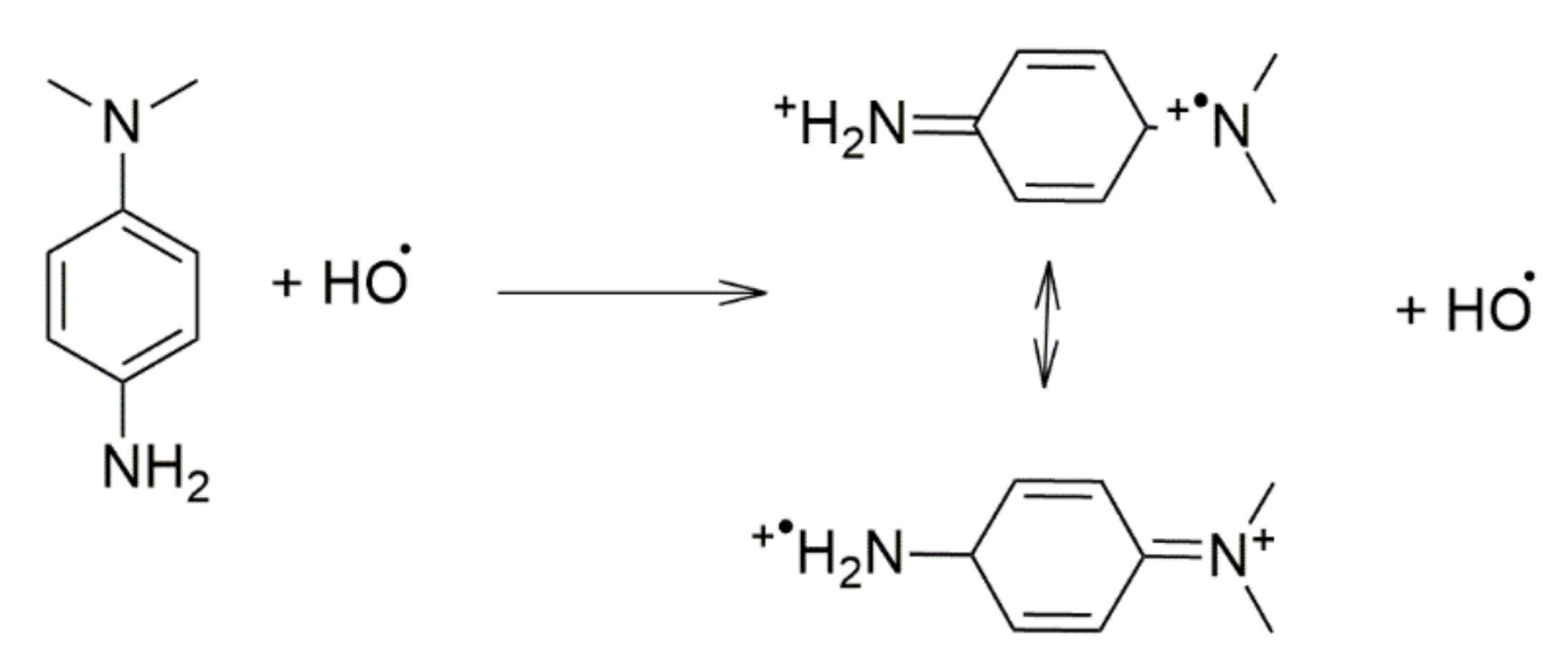
Reaction of *N*,*N*-dimethyl-*p*-phenylenediamine with hydroxyl radical [55].

DMPD radical scavenging assay is based on the conversion of transparent *N*,*N*-dimethyl-*p*-phenylenediamine dihydrochloride (DMPD) into the colored radical DMPD^•+^ in the presence of Fe ions or reactive species, such as hydroxyl radicals (Scheme 3). Antioxidants capable of hydrogen atom transfer decolorize the solution, absorbance decrease at 505 nm [12]. The main disadvantage of using this assay is that DMPD radical is soluble only in water, so there are limitations in using it for hydrophobic antioxidants determination [54].

The FC (Folin-Ciocalteau) assay was initially developed for proteins analysis [56], but later this method was extended by Singleton [57] for the analysis of total phenolic compounds in wine. From chemical point of view, this method is based on the oxidation of phenolic compounds. Folin-Ciocalteau phenol reagent consists of a mixture of phosphomolybdic acid and phosphotungstic acid, in which the molybdenum and tungsten are in the oxidation state 6^+^ and are reduced to the oxidation state of 5^+^ during the reaction. This is accompanied by a color change from yellow original color of the solution of Na_2_WO_4_/Na_2_MO_4_ to blue because of the formation of complexes with phenols (Phenol-MoW_11_O_40_^4−^). The absorbance is usually measured in the range from 750–765 nm [43,44]. This method is mostly used for the determination of total phenolic compounds in various samples (food products, plants, plant extracts), but it can also react in alkaline conditions with non-phenolic compounds, such as amino acids, aromatic amines, ascorbic acid, sulphur dioxide, Cu^+^ ions, and others [43].

**Scheme 4:**
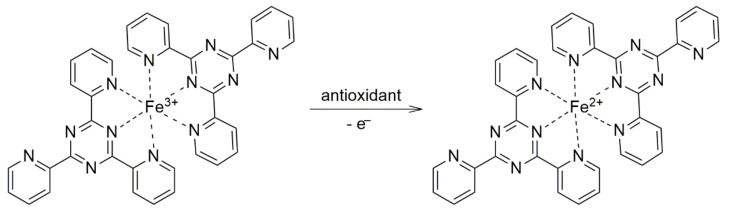
Reduction of Fe^3+^ (FRAP assay) [52].

The FRAP (ferric reducing antioxidant power) assay is based on reduction of colorless Fe^3+^-2,4,6-tripyridyl-*s*-triazine complex to the intensively blue Fe^2+^-2,4,6-tripyridyl-*s*-triazine complex in acidic medium (Scheme 4). FRAP values are calculated from increasing absorbances measured at 593 nm [39,42,43]. The FRAP methods has several limitations. Compounds (even without antioxidant properties) with redox potential lower than the redox potential of Fe^3+^/Fe^2+^ pair may reduce Fe^3+^ to Fe^2+^ and can increase the FRAP value to obtain false high results. But, on the other hand, not all antioxidants reduce Fe^3+^ fast enough to measure [58]. Ferric reducing antioxidant power assay cannot be used for antioxidant acting as radical quench (with transfer of hydrogen) and for compounds absorbing at the wavelength of the determination, as the signals can interfere then [43,58,59].

Oxygen radical absorbance capacity assay (ORAC) is the method for measuring the scavenging activity against peroxyl radicals. The generation of peroxyl radicals have to be induced by AAPH (2,2′-azobis-(2-amidino-propane) dihydrochloride). The antioxidant activity is measured by determining the loss of fluorescence and fluorescein is usually used as a probe [59,60]. This method is popular mainly in the determination of antioxidant ability of foods and is limited by low reactivity of fluorescein and with peroxyl radicals [61].

HORAC (hydroxyl radical averting capacity) technique is based on the oxidation of fluorescein by hydroxyl radicals which are generated using Fenton reagent. Formed antioxidant blocks produced hydroxyl radicals [42,60].

**Scheme 5:**
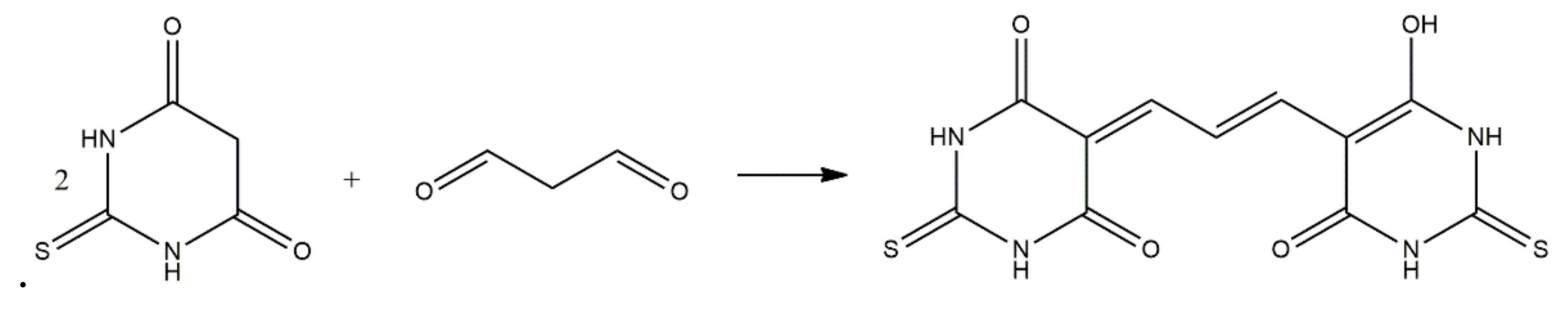
Reaction of two molecules of 2-thiobarbituric acid with malondialdehyde [61].

Other method using the Fenton-like system to induce lipid peroxidation is lipid peroxidation inhibitory assay by measuring the thiobarbituric acid reactive substances (TBARS) [12,42,60]. This assay is based on the reaction of 2-thiobarbituric acid (TBA) with malondialdehyde (MDA) as one of the products of unsaturated lipids oxidation (Scheme 5) [61]. This method is limited by the possibility of MDA reaction with amino group to form Schiff base [62].

Other radical probes, such as TRAP (total peroxyl radical trapping antioxidant parameter) monitoring the reaction between peroxyl radicals and the sample quenched the chemiluminescence, CUPRAC (cupric reducing antioxidant power) by which the reduction of Cu^2+^ is observed, Fremy’s salt (galvinoxyl radical), aroxyl radical (2,6-di-*tert*-butyl-4(4′-methoxyphenyl)phenoxyl radical) [42,60,63,64].

### 3.2. Electrochemical Methods

Electrochemical properties of various compounds can also be used for evaluation of their reducing and antioxidant properties. Electrochemical methods for the determination of total antioxidant capacity are widely used because of their sensitivity, speed, and low cost [65]. Essential electrochemical methods encompass cyclic voltammetry, amperometry, and biamperometry. Cyclic voltammetry (CV) is a method based on the measurement of oxidation potential (E_1/2_) intensity of the sample [43]. The oxidation potential is usually scanned linearly in time from an initial to a final value and back as a triangular waveform. Low values of oxidation potential reflect the tendency of molecule to donate an electron and indicate significant antioxidant capacity [42,43,45,65]. There are some limitations of this method because of the detection limit of 10^−5^ M and low resolution [65]. The amperometric technique involves the measurement of the flowing current intensity between a working and a reference electrode at a fixed value of potential generated by the oxidation-reduction reaction [42,65]. This method is based on the reduction of DPPH• radical at a glassy carbon electrode [65,66,67]. Biamperometry uses two identical polarized platinum electrodes with high sensitivity [42,65,68]. The potential difference (Δ*E*) between the electrodes is controlled and the measurement depends on the reaction of the analyzed sample with redox couple [42,65].

### 3.3. Chromatographic Methods

Chromatography encompasses a group of broadly applicable methods in detection and separation of various compounds present in the analyte, but can also be used for antioxidant capacity measurement. The mostly preferred chromatographic methods are gas chromatography and high-performance liquid chromatography.

High-performance liquid chromatography (HPLC) is used to separate, identify, and quantify the individual components present in the analyte. This method is based on the affinity of analyte to a stationary phase, which is usually non-polar (reverse phase), but also polar (normal-phase) and is placed inside the column. The chosen composition of the mobile phase (eluent) depends on the used stationary phase and interactions between analytes. The separation process depends on affinity of analyte toward the stationary and mobile phases. The analyte flows through column in a liquid mobile phase to a detector (UV/Vis, DAD, MS, etc.,). HPLC is a valuable method for antioxidant capacity determination. The antioxidant properties determination using HPLC system was applied by using on-line post-column detection. First, the separation of sample is promoted and then is mixed with radical solution (ABTS, DPPH). The solution is then directed to a detector and the obtained negative peaks represent a reaction of sample with radical [69,70,71].

### 3.4. Biosensors Method

Biosensors represent analytical tools consisting of a biological recognition element (enzyme, receptor, microorganism, antibody), coupled with a chemical or physical transducer (mass, electrochemical, optical, or thermal) [72]. Potential applications of biosensors for antioxidant capacity determination include monitoring of free radicals, such as nitric oxide (NO), superoxide radical ($O_{2}^{-})$, glutathione (GSH), uric acid, ascorbic acid, or phenolic compounds [72,73].

### 3.5. Nanotechnological Methods

Nanotechnological techniques are usually based on colorimetric total antioxidant capacity assay. The formation of nanoparticles from noble metals, such as gold and silver, by the reaction between metallic salt (Au^3+^, Ag^1+^) and antioxidant leads to the possibility of determination of antioxidant properties of studied samples [12]. When AgNPs are synthesized, the phenomenon of surface-plasmon resonance (SPR) is evident [74,75]. Because of this, metallic nanoparticles (NPs) have interesting attributes such as intensive color which can be detected in visible region of spectra [76]. Reducing compounds are necessary for the formation of nanosuspension, so natural antioxidants are mostly used for nanoparticles preparation and this fact gives the opportunities to detect physicochemical properties including total antioxidant capacity [77]. NPs can be also used for scavenging activity against ROS/RNS determination [77].

## 4. Silver Nanoparticles and Antioxidant Activity Properties

### 4.1. AgNPs Synthesis

Synthesis of Ag nanoparticles that are known mainly for their great antimicrobial activity can be achieved through various methods [78]. In general, we can divide these methods into physical, chemical, and biological. Some of these methods are simple and provide good control of nanoparticle size by affecting the reaction process, but on the other hand, there are still problems with stabilization of the obtained products and to achieve unimodal distribution in nano-region [79]. In Table 2 we show the list of some AgNPs synthesis techniques and several of them are briefly described below.

#### 4.1.1. Physical Methods

Physical approaches for AgNPs synthesis include evaporation-condensation, which has some disadvantages, such as high energy consumption, long time for achieving thermal stability [80,81]. For these reasons, various physical techniques were developed. Pluym et al. used conventional spray pyrolysis [82], Lee and his colleagues synthesized nanoparticles thermal decomposition [83], or ball milling was successfully used by Baláž [84]. This methodology, also called mechanochemical synthesis has recently attracted a significant attention of the research world [85,86,87,88,89,90,91]. Physical methods are useful because of speed and not using toxic chemicals, but on the other hand disadvantages are also there which are previously mentioned [80].

#### 4.1.2. Chemical Methods

Chemical methods represent an easy way to prepare AgNPs in solution. The most common technique for nanoparticles preparation is the reduction by reducing agents of organic or inorganic character, for example sodium ascorbate, hydrogen, *N*,*N*-dimethyl formamide, or sodium borohydride. The principle of AgNPs preparation is the reduction of Ag^+^ ion to metallic form Ag^0^ which is followed by agglomeration into oligomeric clusters leading to the formation of metallic colloidal AgNPs [92]. To avoid the agglomeration of nanoparticles, it is necessary to use stabilizing agents, such as poly(vinyl alcohol), poly(vinyl pyrrolidine), or polyethylene glycol [93,94,95].

#### 4.1.3. Biological Methods

Biological synthesis of nanoparticles in general has become very popular because of its simplicity, low cost, or environmental reasons. The reduction of metallic salt is performed by a natural material including plants, plant extracts, microorganisms, or small biomolecules (amino acids, vitamins or polysaccharides) [96,97,98,99]. According to the used methodology, biological methods can be divided into in vivo and in vitro. In vivo methods use the whole cell for AgNPs biosynthesis, so nanoparticles are synthesized intra- or extracellularly, while during the in vitro process, the reduction of Ag^+^ ions takes places outside of a living organism (the most common are plant extracts containing the compounds with antioxidant and reducing properties—polyphenols, flavonoids, terpenes, aldehydes, carbohydrates, etc.,) [100,101,102]. In addition to plant extract, the extracts of edible mushrooms [103,104,105], extract of microorganisms [106,107,108,109], tissue extract [110], algae extract [111] essential oils [112], and simple biomolecules, such as glucose [113], starch [114], dextrin [115], pectin [99], or cellulose [116] can be used.

The term in vivo describes the biological synthesis of AgNPs inside the living organism. The very first article was published by Gardea-Torresdey et al. [117], who reported the synthesis of AgNPs by living plant alfalfa (*Medicago salvia*). They observed that the alfalfa root is able to absorb silver from agar medium to produce AgNPs. Microorganisms are also capable of producing nanoparticles. Some microorganisms are resistant to metal, so they can survive and grow during the production of NPs, so there is no barrier to use bacteria for AgNPs synthesis [118,119,120]. Fungi also represent a valuable producer of silver nanoparticles because of their capability of metals bioaccumulation [121,122].

### 4.2. AgNPs Antioxidant Properties

Antioxidant capacities of various biological samples, pure chemicals, or isolated compounds are well known. Beside the application of AgNPs in diverse areas, the large number of articles dealing with antioxidant properties of silver nanoparticles have been published spreading in the recent decade. In the Table 3 we have summarized the methods used for silver nanoparticles antioxidant capacity determination together with the methods of preparation during recent years. As it can be seen, the number of publications reporting on biosynthesis (especially using plant extract), has prevailed over any other method of synthesizing AgNPs, which may be justified by simplicity, availability, low cost of this approach (Table 3).

**Figure 2:**
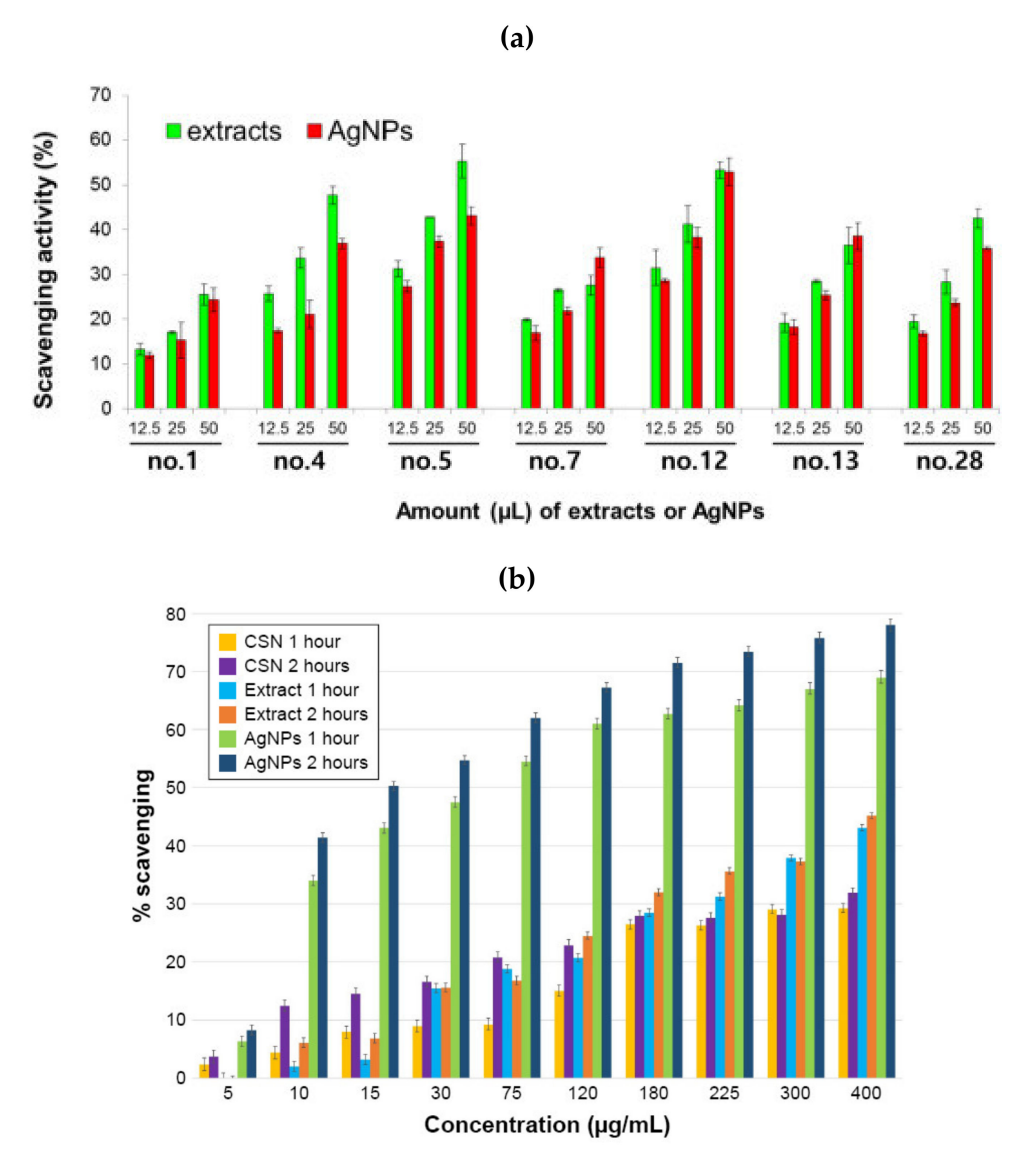
DPPH scavenging activity of (**a**) AgNPs prepared by Chinese plants extract [123], copyright permission by Elsevier; (**b**) AgNPs prepared by walnut green husk extract increasing in concentration and time [124], copyright permission by Dovepress.

From the Table 3 it is also clear that the most commonly used method of AgNPs antioxidant capacity determination is DPPH assay. Authors usually compare the antioxidant capacity of extract with that of prepared silver nanoparticles. In general, contradictory results can be found in literature. For example, Ahn et al. [123] determined antioxidant capacity of thirty plant extracts and AgNPs prepared by using them with the conclusion that the scavenging activity using DPPH assay increased with higher amounts of either extract or AgNPs. In general, the extracts showed better scavenging activity than the AgNPs (Figure 2a). Authors explained this phenomenon by the role of phytochemicals. An extract with high scavenging activity leads to the rapid formation of small AgNPs seeds, which grow into larger nanoparticles with assistance of phytochemicals presented in the matrix [123]. A reduced antioxidant capacity of AgNPs in comparison with extract was also observed by Demirbas [125]. They prepared silver nanoparticles by biological method using extract of red cabbage (*Brassica oleracea* var. *capitate f. rubra*). The authors proposed that AgNPs were synthesized using antioxidant power of red cabbage extract to reduce Ag^+^ ions, so AgNPs may promote superoxide radicals which would consume antioxidant capacity of red cabbage [125]. Lower values of silver nanoparticles (prepared by *Aesculus hippocastanum*) antioxidant capacity was also obtained by Küp [126] who used three different method of scavenging activity determination—DPPH, total reducing power, and superoxide anion radical scavenging assay. The results of DPPH and superoxide anion radical scavenging assays lead to reduced antioxidant capacity of prepared nanoparticles in comparison to plant extract, but the total reducing power of AgNPs indicated more reducing activity than plant extract. The relevant explanation is missing, but authors rely on the fact that reducing power may serve as a major sign of potential antioxidant activity because of the ability of reduction of the Fe^3+^ ferricyanide complex [126].

On the other hand, nanoparticles prepared by Zarrabi et al. [124] showed higher scavenging activity of free radicals by DPPH method than walnut (*Juglans regia*) extract (Figure 2b). They speculated that improved antioxidant properties of AgNPs are due to the simultaneous activity of polyphenols as antioxidant agents and AgNPs as a catalyst [124]. Similar results were noticed by study Elemike et al. [127], who prepared Ag nanoparticles by *Costus afer*. AgNPs showed greater antioxidant capacity than the leaf extract and their activity was comparable to that of ascorbic acid [127]. The present phytochemicals (flavonoids) and silver ions could serve as antioxidants through single electron and hydrogen atom transfer [127]. Other theory was presented by Vijayan et al. [128], who indicate that the increased antioxidant properties of nanoparticles compared to extract can be attributed to the adsorption of bioactive compounds of leaf extract over spherically shaped nanoparticles. Analogically, AgNPs prepared by extract of *Melia azedarach* exhibited higher antioxidant capacity according to extract [129]. According to the authors, the antioxidant ability of AgNPs is caused by the presence of phenolic compounds, terpenoids, and flavonoids in plants which allow nanoparticles to act as singlet oxygen quenchers, hydrogen donors, and reducing agents [129].

An interesting study was published by Das and his co-workers, who studied the antioxidant properties of two types of biosynthesized AgNPs by sweet potatoes *Ipomoea batatas* (L.) [138]. They compared three assays—ABTS, DPPH, and NO_x_ (nitrite/nitrate oxide) at variety of concentrations. The ABTS scavenging activity proceeded in the range of 3.98–12.28%, for DPPH it was 26.30–46.53%, and for NO_x_ the values were 4.30–12.94%. The moderate scavenging effect might arise from the interference of several functional groups presented in extract, which play an important role in the capping and stabilizing on AgNPs. The scavenging activity against DPPH radical is higher than that against ABTS and NO_x_. Lower values of antioxidant capacity against NO_x_ were explained by the difference in the reaction mechanism [138]. Comparison of ABTS and DPPH assay was also published by Otunola [139]. In that study, AgNPs were prepared by garlic (*Allium sativum*), ginger (*Zingiber officinale*), and cayenne pepper (*Capsicum frutescens*) extracts. The AgNPs exhibited potential antioxidant properties against both radicals expressed as IC_50_ values. The highest activity against ABTS was evidenced for cayenne pepper (IC_50_ was 31.25 μg/mL), whereas the highest value of IC_50_ (< 3 1.25 μg/mL) against DPPH radicals was observed for garlic [139].

The DPPH and ABTS assays were also compared by measurements of antioxidant capacity of AgNPs prepared by extract of *Allium ampeloprasum* L. [137]. The antioxidant capacity was determined at different concentrations (100, 200, 300, 400 and 500 mM). The DPPH and ABTS radical scavenging abilities were dose-dependent, which means that increasing scavenging activities against both radicals with the increasing concentration of AgNPs were observed (for DPPH and ABTS the antioxidant activity was in the range 62.2–82.4% and 64.5–96.8%, respectively). These results can be considered slightly inadequate, because the authors did not compare the antioxidant capacity with that of standard, so it can be only hypothesized that silver nanoparticles prepared by using the extract of *Allium ampeloprasum* L. exhibit good antioxidant activity [137].

In addition to plant extract, bacterial strains are also used for AgNPs biosynthesis. As an example, the study on the use of *Trichoderma atroviride* for this purpose can be mentioned [153]. The presented work showed that AgNPs exhibited quite higher DPPH scavenging activity in a concentration-dependent manner with IC_50_ of 45.6 μg/mL [153].

**Figure 3:**
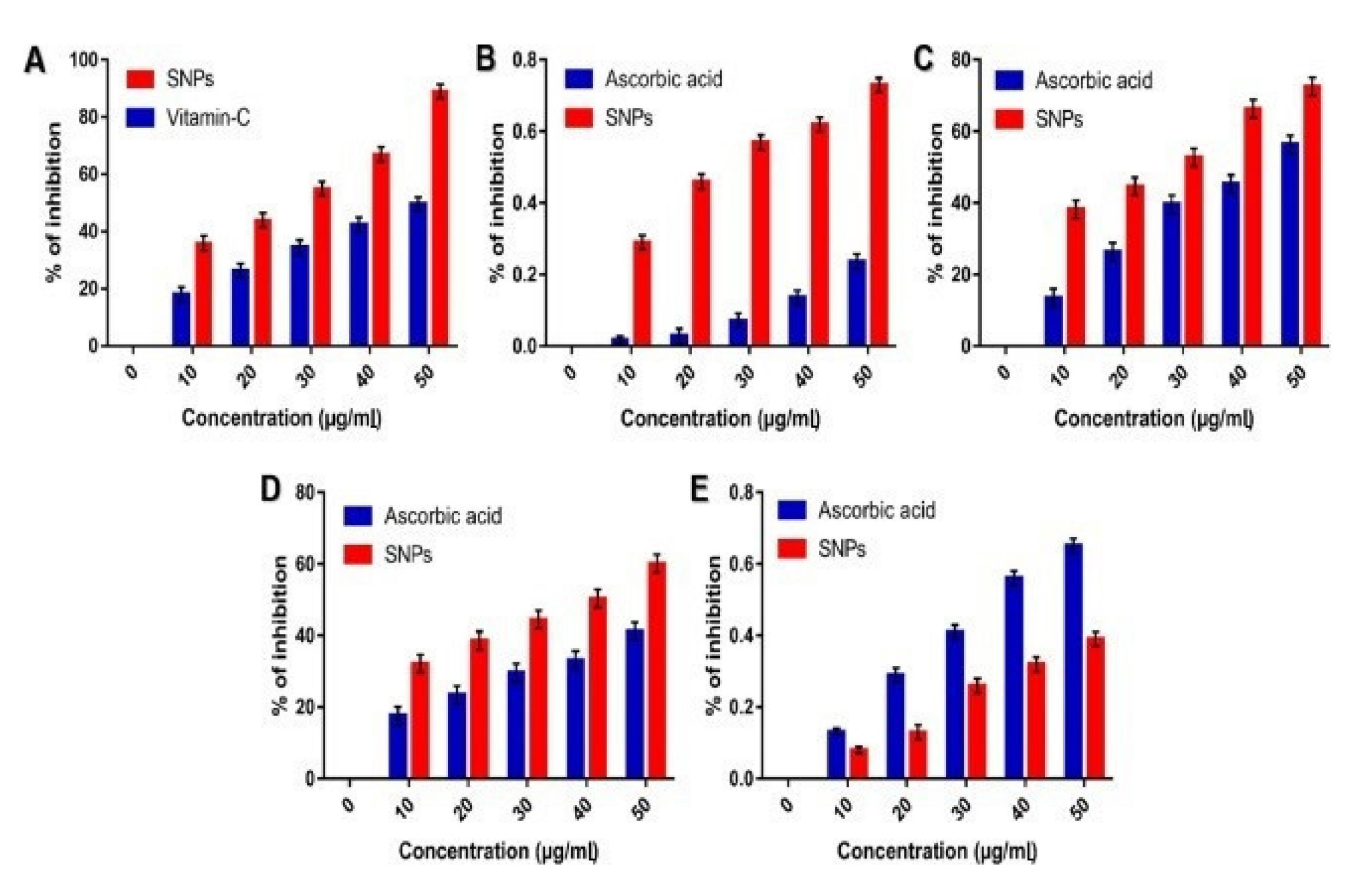
(**A**) DPPH assay, (**B**) total antioxidant activity, (**C**) H_2_O_2_ scavenging activity_,_ (**D**) nitric oxide scavenging activity, (**E**) ferric reducing power assay of *S. violaceus* MM72 exopolysaccharide-mediated AgNPs with their respective standards. The data represent mean ± SD of the three replicates (*n* = 3) [155], copyright permission by Elsevier.

Biomolecules isolated from microorganisms, such as exopolysaccharides were also used for biosynthesis of AgNPs [154,155]. Promising antioxidant properties were reported by Sivasankar et al. [155], who studied antioxidant capacity of silver nanoparticles prepared by exopolysaccharides isolated from *Streptomyces violaceus*. They focused on DPPH, NO, and H_2_O_2_ scavenging activities, total antioxidant activity, and ferric reducing power assay using ferricyanide (Figure 3). The AgNPs showed the DPPH radical scavenging effect of 89.5% at concentration of 50 μg/mL, which was higher than in the case of ascorbic acid standard (49.6%). Analogically, they found higher total antioxidant activity of AgNPs compared to the standard. Hydrogen peroxide scavenging activity of the studied AgNPs (72.5 %) was also higher than standard (56.4%). The authors explained the results based on the hypothesis that in the presence of H_2_O_2_ the dispersed nanoparticles may induce the formation of reactive oxygen species and hydrogen peroxide inside a cell. Then AgNPs yield greater amounts of hydrogen peroxide and induce inflammasome formation leading to the production of superoxide and H_2_O_2_ in the mitochondria’s membrane [155]. Nitric oxide assay also showed a significant activity of AgNPs (60.1%) being higher than that of L-ascorbic acid (41.2%). Ferric reducing power of AgNPs was lower than that of ascorbic acid standard and was higher at lower concentrations of AgNPs. These findings make the silver nanoparticles useful for neurodegenerative diseases, cancer, or AIDS treatment [155,162].

Similar results were published by Shahid et al. [154] who determined antioxidant properties of silver nanoparticles prepared by exopolysaccharides from *Lactobacillus brevis*. The focus was on nitric oxide, DPPH, and hydrogen peroxide radical scavenging activity. The authors observed excellent nitric oxide scavenging activity of AgNPs in a concentration-dependent manner, but the activity was in general lower than that of ascorbic acid standard. At concentration of 100 μg∙mL^−1^ they determined the activity to be 75.06 ± 0.4%, whereas for ascorbic acid, it was 91.1 ± 1.5%. Hydrogen peroxide and DPPH scavenging activity of AgNPs was also compared with ascorbic acid. On the contrary to NO_x_ assay, the ability to scavenge both free radicals of silver nanoparticles was higher than that of the standard used. Namely, for AgNPs with concentration of 100 μg∙mL^−1^ the activity of H_2_O_2_ scavenging was 70.1 ± 0.6% and in the case of DPPH assay it was 91.3 ± 0.7%. The ascorbic acid standard showed 55.0 ± 0.7% activity for hydrogen peroxide technique and 81.4 ± 1.2% for DPPH radical scavenging activity [154].

The free radical scavenging activity of *Teucrium polium* extract, and of AgNPs produced chemically and by green synthesis was studied by DPPH and FRAP assays in [163]. The results show that the activity of green synthesized silver NPs increased with higher concentration, while that of chemically synthesized nanoparticles did not show a significant antioxidant activity. The antioxidant capacity of green-synthesized AgNPs was similar to that of plant extract. The authors explained these results by a suggestion that the presence of bioactive compounds on the surface of AgNPs is responsible for the majority of antioxidant capacity and silver nanoparticles are not contributing much [163].

DPPH method was used to determine free radical scavenging activity of silver nanoparticles prepared chemically (using sodium citrate served as reducing agent) and biologically (using extract of *Datura stramonium* plant as a reducing agent) in [149]. The DPPH radical scavenging ability of green and chemically prepared AgNPs was compared with that of plant extract. Better results were observed for green-synthesized silver nanoparticles [149].

Mittal [157] and his co-workers published a study dealing with the synthesis of AgNPs using *Syzygium cumini* extract. They observed that the reduction of silver ions and stabilization of prepared AgNPs were mainly because of the action of flavonoids. The authors evaluated the antioxidant properties by Folin-Ciocalteau’s assay for total phenolic and flavonoid content, DPPH, ABTS, and MTT methods. The total phenolic content was lower (8.1 ± 0.1%) than the total flavonoid content (81.2 ± 0.27%), so they concluded that the amount of flavonoids present in silver nanoparticles was ten times higher which was in good agreement with the isolated flavonoids from extract. The DPPH assay showed antioxidant capacity of 59% (in comparison to 87% detected by Trolox at the same concentration). On the other hand, upon using ABTS method, they observed 63% scavenging activity and when using MTT test, 61% scavenging activity at the same concentration (50 μg∙mL^−1^) was observed [157].

Various phenolic compounds including flavonoids (quercetin, rutin, hesperidin), benzoic acids (gallic acid, protocatechuic acid sylicylic acid, benzoic acid), and cinnamic acids (caffeic acid, ferulic acid, *p*-coumaric acid and *trans*-cinnamic acid) were used for silver nanoparticles synthesis in [161]. The antioxidant capacities of structurally different phenolic compounds were evaluated. The hydroxylation of aromatic ring played an important role in the reactivity to form silver NPs. The higher degree of hydroxylation in chemical structures of phenolic compounds demonstrated the higher radical scavenging capacity and higher tendency to reduce Ag^+^ to AgNPs [161].

(PABA-PVA) AgNPs were subjected to the ABTS, DPPH, and H_2_O_2_ radical scavenging activities measurements in [160]. All tested nanoparticles have exhibited lower scavenging activity than ascorbic acid standard. The highest free radical scavenging activity was determined against DPPH radical followed by H_2_O_2_ and ABTS methods [160].

The expression of antioxidant activity only as a percentage of inhibition has little significance, as antioxidant activity is strongly dependent on conditions, such as solvent and concentration of radicals. In any case, it is better to express or compare the activity with a standard (ascorbic acid, trolox, gallic acid, quercetin, etc.). When comparing the activity with the extract, the choice of concentration seems to be a key factor influencing the result, as the antioxidant activity is concentration-dependent. The resulting antioxidant activity of the nanoparticles significantly depends mainly on the reducing substances in the extract bounded/capped to the surface of the nanoparticles.

For the basic study of antioxidant activity, the use of various in vitro methods is a suitable choice, either with a mechanism of action such as hydrogen transfer or electron transfer. In these methods, the mechanism of antioxidant action of silver nanoparticles can be ascribed to the fact that silver can exist in two oxidation states (Ag^+^ and Ag^2+^) depending on the reaction conditions and the produced AgNPs may be able to quench free radicals by donating or accepting electrons [164]. Nanoparticles have been shown to have a more complicated mechanism of action in biological systems using in vivo activity-based methods.

## 5. Conclusions

This brief review provided an overview on the antioxidant activity of silver nanoparticles and the methods of its measurement. Generally, there are three methods for AgNPs synthesis—biological, physical, and chemical. Obviously, the most commonly used synthesis approach to AgNPs synthesis is the biological one, which utilizes mainly plant extracts to reduce silver ion to form and stabilize the Ag^0^ nanoparticles. This approach is becoming more popular because of the environmental and economic reasons.

The antioxidant properties of AgNPs are usually evaluated by DPPH and ABTS free radical scavenging assays. As the most established nanoparticles are synthesized using plant extracts, the antioxidant capacity of AgNPs is often compared with plant extract itself. The results are contradictory, as some authors observed higher antioxidant capacity of AgNPs, whereas on the other hand, there are also studies with the opposite results. Based on the large number of papers reporting both types of results, it seems that both scenarios are possible. In general, the antioxidant properties of silver nanoparticles depend on the chemical composition of the extract and it usually improves with the increase of the AgNPs concentration. If the extract is rich in phenolic compounds and flavonoids, the nanoparticles exhibit high scavenging activity.

This review has shown that there are many methods used for antioxidant capacity determination. It is hard to conclude which method is the most suitable because of various composition of nanoparticles synthesized by biological methods depending on the phytochemicals presented in plant extracts. The antioxidant test models vary in different aspects. Therefore, the total antioxidant capacity cannot by evaluated on the basis of a single antioxidant test model and it is also difficult to compare one method with another. In the present state, the use of combination of different methods is always beneficial. However, understanding the roles of various antioxidants and their activities is challenging. Because of this, there is an enormous need to develop a uniform protocol for determining the antioxidant capacity of nanoparticles to precisely assess their potential.

## Figures and Tables

**Table 1:** Methods for antioxidant capacity evaluation.

Method	Principle	Final Product Determination
Spectrometric		
DPPH	Reaction with organic radical	Colorimetry
ABTS	Reaction with organic radical	Colorimetry
DMPD	Reaction with organic radical	Colorimetry
FC	Reaction with Mo^6+^ and W^6+^	Colorimetry
FRAP	Reaction with Fe^3+^	Colorimetry
ORAC	Reaction with peroxyl radical initiated by AAPH	Fluorescence loss
HORAC	Reaction with OH radicals generated by Co^2+^ based Fenton-like systems	Fluorescence loss
TRAP	Reaction with luminol-derived radicals, generated during the AAPH decomposition	Quench of chemiluminescence
Lipid peroxidation inhibitory assay	Fenton-like system (Co^2+^ + H_2_O_2_)	Colorimetry
PFRAP	Potassium ferricyanide reduction	Colorimetry
CUPRAC	Cu^2+^ reduction to Cu^1+^	Colorimetry
Fluorimetry	Emission of light by a substance that has absorbed the light or other electromagnetic radiation of a different wavelength	Fluorescence excitation/emission spectra
Electrochemical		
Cyclic voltammetry	The potential of working electrode is varying from initial to final value and back, current intensity is recorded	Measurement of the intensity of cathode or anode peak
Amperometry	The potential of working electrode is fixed to a reference electrode	Measurement of the intensity currently produced by oxidation or reduction of a sample
Biamperometry	The reaction of an antioxidant with the oxidized form of a reversible redox couple	Measurement of the current flow between two identical working electrodes
Chromatographic		
High performance liquid chromatography	Separation of compounds in a reaction mixture at a stationary phase in a liquid mobile phase	UV/Vis, MS or fluorescence detection
Biosensors	Enzyme-based biosensors measuring total phenolic content	Electroanalytical evaluation
Nanotechnological methods	Reaction of noble metal (Au, Ag) salt with antioxidant compound	Colorimetry

**Table 2:** Selected techniques for AgNPs preparation.

Silver Nanoparticles Synthesis			
Physical Methods	Chemical Methods	Green Synthesis Methods	
		In Vitro Methods	In Vivo Methods
Arc discharge Ball milling Evaporation–condensation Pulsed laser ablation Spray pyrolysis Vapor and gas phase	Electrochemical Microwave assisted Photochemical Reduction Sonochemical	Using algae Using biomolecules Using essential oils Using microorganisms Using mushroom extracts Using plant extracts	Using algae Using microorganisms Using plant Using yeast

**Table 3:** Methods of antioxidant capacity determination of prepared silver nanoparticles.

Method of Antioxidant Capacity Measurement	Method of AgNPs Synthesis	Reducing Agent	Precursor	Ref.
DPPH	Biological	Plant extracts of *Cratoxylum formosum*, *Phoebe lanceolata*, *Scurrula parasitica*, *Ceratostigma minus*, *Mucuna birdwoodiana*, *Myrsine africana* and *Lindera strychnifolia*	0.25 mM AgNO_3_	[123]
DPPH	Biological	Walnut (*Juglans regia*) green husk extract	6 mM AgNO_3_	[124]
DPPH	Biological	Plant extract of *Costus afer*	1 mM AgNO_3_	[127]
DPPH	Biological	Plant extract of *Indigofera tinctoria*	1 mM AgNO_3_	[128]
DPPH	Biological	Red cabbage (*Brassica oleracea* var. *capitate f. rubra*) extract	5 mM AgNO_3_	[125]
DPPH	Biological	Plant extract of *Clerodendrum inerme*	1 mM AgNO_3_	[130]
DPPH	Biological	Plant extract of *Rhododendron dauricum*	0.5–5 mM AgNO_3_	[131]
DPPH	Biological	Plant extract of *Ougeinia oojeinensis*	1 mM AgNO_3_	[132]
DPPH FC method	Biological	Plant extract of *Bergenia ciliata*	0.1% AgNO_3_	[133]
DPPH FRAP	Biological	Plant extract of *Prosopis farcta*	1 mM AgNO_3_	[134]
DPPH ABTS •OH Superoxide anion NO	Biological	Plant extract of *Pongamia pinnata*	1 mM AgNO_3_	[135]
DPPH ABTS	Biological	Plant extract of *Tropaeolum majus*	1 mM AgNO_3_	[136]
DPPH ABTS	Biological	Plant extract of *Allium ampeloprasum* L.	1 mM AgNO_3_	[137]
DPPH ABTS NO_x_	Biological	Peels’ extract of *Ipomoea batatas* (L.)	1 mM AgNO_3_	[138]
DPPH ABTS	Biological	Plant extracts of *Allium sativum*, *Zingiber officinale*, *Capsicum frutescens*	0.1 M AgNO_3_	[139]
DPPH ABTS NO_x_	Biological	Plant extract of *Taraxacum officinale*	1 mM AgNO_3_	[140]
DPPH FRAP	Biological	Plant extract of *Teucrium polium*	3 mM AgNO_3_	[141]
DPPH	Biological	Plant extract of *Pulicaria vulgaris*	1 mM AgNO_3_	[142]
DPPH	Biological	Plant extract of *Gynura procumbens* encapsulated with fungal chitosan from *Cunninghamella elegans*	1 mM AgNO_3_	[143]
DPPH Reducing power Superoxide anion	Biological	Plant extract of *Aesculus hippocastanum*	5 mM AgNO_3_	[126]
DPPH FC Superoxide anion	Biological	Plant extract of *Indian belladonna*	1 mM AgNO_3_	[144]
DPPH ABTS	Biological	Plant extract of *Melia azedarach*	1 mM AgNO_3_	[129]
DPPH ABTS •OH Superoxide anion H_2_O_2_	Biological	Extract of garlic (*Allium sativum*), green tea (*Camellia sinensis*), turmeric (*Curcuma longa*)	25 mM AgNO_3_	[145]
DPPH ABTS	Biological	Plant extract of *Psidium guajava L.*	1 mM AgNO_3_	[146]
DPPH FRAP	Biological	Plant extract of *Bauhinia variegata*	1 mM AgNO_3_	[147]
DPPH	Biological	Plant extract of *Achillea millefolium*	1 mM AgNO_3_	[148]
DPPH	Biological	Plant extract of *Datura stramonium*	1 mM AgNO_3_	[149]
DPPH ABTS	Biological	Corn (*Zea mays*) flour extract	1 mM AgNO_3_	[150]
Superoxide H_2_O_2_ NO DPPH •OH	Biological	Essential oil of *Coleus aromaticus*	0.214 mM AgNO_3_	[151]
DPPH FC	Biological	Macroalgae *Ulva lactuca* L.	5 mM AgNO_3_	[152]
DPPH	Biological	Bacterial strain *Trichoderma atroviride*	5 and 10 mM AgNO_3_	[153]
DPPH H_2_O_2_ NO	Biological	Exopolysaccharide from probiotic *Lactobacillus brevis*	2 mM AgNO_3_	[154]
DPPH H_2_O_2_ NO Ferric reducing power assay	Biological	Exopolysaccharide from *Streptomyces violaceus*	3 mM AgNO_3_	[155]
DPPH	Biological	*Ochrobactrum rhizosphaerae*	9 mM AgNO_3_	[156]
DPPH ABTS MTT FC	Biological	*Syzygium cumini* fruit extract	0.5–5 mM AgNO_3_	[157]
DPPH	Chemical	Sodium citrate	45 mg AgNO_3_ Stabilized by PVP or PVA	[158]
TAC TBARS PCARB GSH	Chemical	EG EG/PVP	AgNO_3_	[159]
DPPH ABTS H_2_O_2_	Chemical	PABA-PVA	0.1 M AgNO_3_NaOH	[160]
DPPH	Chemical	PLA/PEG	1 mM AgNO_3_	[149]
DPPH ABTS	Chemical	Phenolic compounds	AgNO_3_ Stabilized by PVP NaOH	[161]

EG—ethylene glycol; EG/PVP—ethylene glycol/poly(vinyl pyrollidine); PABA-PVA—poly(3-aminophenyl boronic acid- poly(vinyl alcohol); PLA/PEG—poly lactic acid/poly ethylene glycol.
